# Traditional Chinese Medicine and Vascular Disease

**DOI:** 10.1155/2015/430818

**Published:** 2015-10-21

**Authors:** Yanwei Xing, Dan Hu, Tan Zhang, Charles Antzelevitch

**Affiliations:** ^1^Department of Cardiology, Guang' anmen Hospital, China Academy of Chinese Medical Sciences, Beijing 100053, China; ^2^Experimental Cardiology and Molecular Genetics, Masonic Medical Research Laboratory, Utica, USA; ^3^Department of Internal Medicine, Gerontology and Geriatric Medicine, Wake Forest School of Medicine, Medical Center Boulevard, Winston-Salem, NC, USA

According to the epidemiological survey, vascular disease is the leading cause of death in the world. Vascular disease includes any condition that affects your circulatory system, such as peripheral artery disease. This ranges from diseases of your arteries, veins, and lymph vessels to blood disorders that affect circulation. With the world population aging, hypertension, stroke, coronary heart disease, and diabetes incidences are increasing year by year. These diseases seem to be independent; in fact, vascular disease is able to cause all these diseases. However, the recent studies suggest that traditional Chinese medicine (“TCM”) is a potential candidate for the preventative treatment of vascular disease. For example, Tongxinluo capsule for the treatment of atherosclerosis has been recognized around the world.

TCM is an integral part of Chinese culture, which includes pharmacology of traditional Chinese medical formulae, Chinese patent medicines, and Chinese herbal monomer. Today both of TCM and western medicine are being used in providing medical and health services in the whole world. TCM, with its unique diagnostic methods, systematic approach, abundant historical literature, and materials, has attracted many attentions from the international community. TCM has been very effective in the treatment of vascular disease, but it still has the following problems. (1) Many mechanisms of TCM for diseases are not clear. (2) Large-scale clinical trials based on evidence are lacking. Therefore, we encourage investigators to contribute original research articles as well as comprehensive review articles on the treatment of vascular diseases with TCM. We are particularly interested in articles reporting the clinical trials or revealing the action mechanisms of TCM by using cellular or animal models.

*Cerebral Vascular Related Articles*. X. Han et al. in their paper “The Clinical Relevance of Serum NDKA, NMDA, PARK7, and UFDP Levels with Phlegm-Heat Syndrome and Treatment Efficacy Evaluation of Traditional Chinese Medicine in Acute Ischemic Stroke” found that the serum PAPK7 and UFDP concentration exhibited diagnostic value for the phlegm-heat syndrome of TCM in patients. The serum NDKA, NDMA, PAPK7, and UFDP levels did not show a significant tendency of increase in day 7 compared to 3 days within onset, but UFDP, but not NDKA levels, exhibited a tendency of increase in day 14 compared to day 7.

X. Du et al. in their paper “Scutellarin Reduces Endothelium Dysfunction through the PKG-I Pathway” investigated the protective mechanism of scutellarin (SCU) in vitro and in vivo for human brain microvascular endothelial cells (HBMECs). SCU protects against cerebral vascular EtD through endothelial PKG pathway activation.

*Mechanism of Chinese Herbal Medicine on the Protection of Renal Vascular*. B. Liu et al. in their paper introduced to us the protective effects of curcumin on obesity-related glomerulopathy. Curcumin is able to alleviate the harmful reaction of leptin on podocytes and reduce the severity of ORG. The above protective effects are associated with the inhibition of Wnt/catenin signaling activation in podocytes.

L. Han et al. in their paper “The Renal Protective Effect of Jiangya Tongluo Formula, through Regulation of Adrenomedullin and Angiotensin II, in Rats with Hypertensive Nephrosclerosis” make a conclusion that Jiangya Tongluo Formula can prevent nephrosclerosis through regulation of adrenomedullin and angiotensin II. JYTL may upregulate endogenous ADM level in the kidneys and antagonize Ang II during vascular injury by dilating renal blood vessels and improving ischemia, thus resulting in the protection of renal function.

*Mechanism of Chinese Herbal Medicine on the Protection of Diabetic Vascular Disease*. M. Li et al. in their paper “Tang-Tong-Fang Confers Protection against Experimental Diabetic Peripheral Neuropathy by Reducing Inflammation” have studied Chinese herbal medicine in the protection of diabetic vascular disease. TTF treatment also attenuated the effect of DPN on other parameters including histology and ultrastructural changes, expression of ICAM-1, MPO, and TNF-*α* in rat sciatic nerves, and plasma sICAM-1 and MPO levels. Together, our data suggest that TTF treatment may alleviate DPN via ICAM-1 inhibition.

*Mechanism of Chinese Herbal Medicine Treatment on Atherosclerosis*. Q. Kang et al. in their paper “Effect of Compound Chuanxiong Capsule on Inflammatory Reaction and PI3K/Akt/NF-*κ*B Signaling Pathway in Atherosclerosis” suggested that Compound Chuanxiong Capsule can prevent atherosclerosis through regulation of the inflammatory reaction and PI3K/Akt/NF-*κ*B signal pathway. They concluded that CCC can inhibit inflammatory reaction in the ApoE−/−mice fed with a high fat diet. Its mechanism may be related to regulation of PI3K/Akt/NF-*κ*B signaling pathway.

S.-H. Lu et al. in their paper “Experimental Study of Antiatherosclerosis Effects with Hederagenin in Rats” studied that hederagenin had the effect of antiatherosclerosis. The results indicated that hederagenin can inhibit or improve the pathological changes that occur during atherosclerosis induced by a high-fat diet plus VD3 in rats. The underlying mechanism might be related to the regulation of lipid metabolism disorders, improvement of blood rheology, regulation of vascular endothelium imbalance, and inhibition of the IKK*β*/NF-*κ*B signaling pathway to reduce the amplification cascade of the inflammatory response.

M. Wu et al. in their paper “Polydatin Inhibits Formation of Macrophage-Derived Foam Cells” have studied that polydatin protected against atherosclerosis through inhibiting formation of macrophage-derived foam cells. Polydatin significantly inhibits the formation of foam cells derived from peritoneal macrophages. Further studies indicated that polydatin regulates the metabolism of intracellular lipid and possesses anti-inflammatory effects, which might be regulated through the PPAR-*γ* signaling pathways.

The accepted articles mainly introduced TCM which included pharmacology of traditional Chinese medical formulae, Chinese patent medicines, and Chinese herbal monomer, which protected against atherosclerosis through improving the vascular endothelial function, inhibiting the oxidative stress, and suppressing inflammatory reaction. In this special issue, the articles included are not only basic research, but also clinical research. The diseases to be intervened included diabetic vascular disease, cardiovascular disease, cerebrovascular disease, peripheral vascular diseases, microvascular diseases, and renal vascular diseases. TCM treatment of the vascular disease may be an effective method.

*Yanwei Xing* *Yanwei Xing* *Dan Hu* *Dan Hu* *Tan Zhang* *Tan Zhang* *Charles Antzelevitch* *Charles Antzelevitch*

